# Case of Dislocation of the Scaphoid Bone of the Foot

**Published:** 1851-03

**Authors:** 

**Affiliations:** Peterborough


### SURGERY.

*Case of Dislocation of the Scaphoid Bone of the Foot.* By Dr. Walker, Peterborough.—Dislocation of the scaphoid or navicular bone of the tarsus is an accident of very rare occurrence; the position and the connexions of this bone render its displacement so improbable, that it is generally regarded as impossible that it should occur, "unless," as Mr. B. Cooper remarks in his "Treatise on the Ligaments," "it be attended with total destruction of the foot." To the extensive observation of Sir A. Cooper no such case had ever presented itself; the following therefore must be regarded as one of peculiar interest:—

On the 10th of June, 1850, a stone-mason, aged 40, and of a spare but sinewy form, in stepping from the top of a wall, eighteen feet high, to another about a foot lower, over an intervening space of about two feet, advanced his right foot, but stepped short, his toes only resting upon the wall, his whole weight being, in the act of stepping, thrown upon the right leg. The foot, as he says, "bent upwards, and something gave way." Falling down between the two walls, he found himself on the ground on his hands and knees, not hurt, as he conceived, by the fall, but quite unable to work, and his foot in great pain. He was carried home, and I saw him next day. He was in bed, and on examination my opinion of the case entirely coincided with that of Mr. Hanbury, our excellent house-surgeon, who had previously visited him. We found a bold projection on the dorsum pedis, in the situation of the os naviculare, and a corresponding depression beneath, where, in the normal condition, the tuberosity of this bone forms a distinct projection. Over the displaced bone the skin was tense and red, and over this point there was a good deal of pain on pressure. He could bend and extend the ankle-joint without difficulty or much increase of pain. I at first tried to reduce the bone by firm pressure with the thumbs, but it felt firmly fixed in its abnormal position, and I could not move it.

I then requested Mr. Hanbury to press the foot downwards, whilst I attempted to return the bone to its position between the astragalus and cuneiform bones, and with an audible snap it returned, and the deformity disappeared.

I then merely directed the foot to be kept quiet and fomented; but two days after, finding that the bone felt loose and easily moveable, with a tendency to slip upwards, occasioning the peculiar appearance of a slight dimple or hollow in place of its natural projection, a plaster and roller were applied.

He was confined to the house for three weeks. He then returned to his work, but continued slightly lame for about a month after. This morning (Sept. 16th) I find, on examination, no remaining appearance of injury; he has no pain, and the motions of the foot are perfect.

The practical conclusions to be drawn from this case are, that although rare, this dislocation is not impossible; that when it does occur it is not difficult of detection, neither is there any peculiar difficulty in its reduction; also, that in the absence of any complication from more extensive injury to the structure of the tarsus, than *in this case must have existed*, we may venture to anticipate complete recovery, notwithstanding the injury to, and probable laceration of, the astragalo-scaphoid and calcaneo-scaphoid ligaments, as well as of the tendon of the tibialis posticus muscle, which are the necessary conditions of such a complete dislocation of the scaphoid bone as I have here described.—*Prov. Med. & Surg. Journal.*

